# MicroRNAs as regulators of tumor-associated stromal cells

**DOI:** 10.18632/oncotarget.1625

**Published:** 2013-11-21

**Authors:** Brian Ell, Yibin Kang

**Affiliations:** Department of Molecular Biology, Princeton University, NJ USA; Department of Molecular Biology, Princeton University, NJ USA

The interplay between tumor and stromal cells represents an essential component of tumor progression and metastasis, and has become a focal point of current research efforts. Tumor-stromal interactions support early events in tumorigenesis, mediate the formation of pre-metastatic niches, and regulate the initiation and progression of distant metastases. Developing a better understanding of stromal regulation will be necessary for future therapeutic strategies, although progress has traditionally been hindered due to the complexity of the stromal microenvironment. Despite this, multiple studies have now examined the regulation of stromal microRNAs (miRNAs) during tumor progression, revealing the potential for miRNA-based therapeutic strategies to combat metastasis.

We have recently described broad miRNA expression changes in osteoclasts, essential bone-resorbing stromal cells, during bone metastasis (Ell et al, *Cancer Cell*, 2013, 24;542-556). Osteoclastic activity drives lytic breast cancer bone metastasis, degrading bone and releasing bone-derived growth factors that enhance tumor growth. Thus, inhibiting osteoclast differentiation, through the blockage of osteoclast differentiation factors such as RANKL, or via direct inhibition with bisphosphonates, has proven to be a reliable method of limiting bone metastasis progression. Paramount within our study was the discovery that tumor-secreted factors could induce broad miRNA changes within osteoclasts, mimicking miRNA changes seen in physiological osteoclast differentiation. Mechanistically, osteoclast miRNA changes were induced through downstream signaling from soluble ICAM1 and other tumor-secreted factors. Importantly, specific ectopic expression of miR-141 or miR-219 was capable of inhibiting osteoclast differentiation and limiting bone metastasis in a mouse model of breast cancer metastasis. A similar study (Squadrito et al, *Cell Reports*, 2012, 1;141-154) discovered activity of miR-511-3p in tumor-associated macrophages (TAMs), well known mediators of tumor progression. The authors discovered that ectopic expression of miR-511-3p reduced the pro-tumoral activity of TAMs and inhibited the growth of Lewis lung carcinoma in mice. Interestingly, a comparable study has revealed a miRNA signature capable of recapitulating a cancer-associated fibroblast (CAF) phenotype in normal omental fibroblasts (Mitra et al, *Cancer Discovery*, 2012, 2;1100). Normal fibroblasts transfected with anti-miR-31, anti-miR-214, and miR-155 displayed enhanced migration, as well as elevated invasion and colony formation of co-cultured ovarian cancer cells, and increased tumor growth *in vivo*. Importantly, in an opposite set of experiments, elevating expression of miR-31 and miR-214 while inhibiting miR-155, abrogated the CAF phenotype. Together, these studies reveal the potential for utilizing miRNAs to target the stromal compartment and thereby limit tumor progression.

An additional discovery within our study was the capability to utilize serum levels of miRNAs as biomarkers for bone metastasis progression in patients. It is currently difficult to predict which patients will develop bone metastasis, and standard biomarkers, such as N-terminal telopeptide, show only modest specificity as diagnostic markers. Interestingly, we found that two miRNAs, miR-16 and miR-378, were elevated in bone metastasis patients relative to healthy donors. It is not currently known exactly how miRNAs are secreted into the serum, although it has been hypothesized that miRNA trafficking could occur through the secretion of exosomes. While the miRNAs examined in our study did not show an overt effect on osteoclast differentiation or tumor cell proliferation, it is possible that secreted miRNAs can be laterally transferred between stromal and tumor cells, leading to enhanced metastatic potential. Indeed, TAM secreted miR-223 can enhance breast cancer invasiveness *in vitro*, and might indicate a novel mechanism of communication between macrophages and tumor cells (Yang et al, *Molecular Cancer* 2011, 10;117).

These studies raise a number of interesting questions in regard to the therapeutic potential for miRNAs. Can miRNAs be useful as therapeutics, not only as direct inhibitors of metastatic cells, but also to disrupt tumor-stromal interactions? These preliminary studies show promising results, although rigorous follow-up studies will be necessary before advancing to clinical trials. Additionally, our preliminary results suggest a potential additive effect when miRNAs were combined with the bisphosphonate Zometa. It will be interesting to examine combinatorial treatments with miRNAs and current antiresorptive therapies, as well as the usefulness of miRNAs as inhibitors of other diseases associated with pathological osteoclast activity, including Paget's disease and osteoporosis. Beyond osteoclasts, can miRNAs be useful as inhibitors of other stromal cells, including TAMs, fibroblasts, and endothelial cells, and will these treatments be effective in limiting metastasis within their respective tumor models? Finally, a major concern within miRNA treatments involves off-target effects, and it will be essential to confirm specificity and low risk of side effects as development of these therapeutics progresses.

In summary, these studies reveal the potential for utilizing miRNAs as therapeutic targets to limit the activity of tumor-associated stromal cells. In addition, secreted stromal miRNAs might prove useful as functional mediators of metastatic progression or clinical biomarkers. While a number of questions remain as to the utility, efficacy, and safety of miRNA-based therapeutic strategies, we hope that our efforts will facilitate the development of novel treatments for metastasis.

